# Limbic-thalamo-cortical projections and reward-related circuitry integrity affects eating behavior: A longitudinal DTI study in adolescents with restrictive eating disorders

**DOI:** 10.1371/journal.pone.0172129

**Published:** 2017-03-01

**Authors:** Gaia Olivo, Lyle Wiemerslage, Ingemar Swenne, Christina Zhukowsky, Helena Salonen-Ros, Elna-Marie Larsson, Santino Gaudio, Samantha J. Brooks, Helgi B. Schiöth

**Affiliations:** 1 Department of Neuroscience, Functional Pharmacology, Uppsala University, Uppsala, Sweden; 2 Department of Women's and Children's Health, Uppsala University, Uppsala, Sweden; 3 Department of Neuroscience, Child and Adolescent Psychiatry, Uppsala University, Uppsala, Sweden; 4 Department of Surgical Sciences, Radiology, Uppsala University, Uppsala, Sweden; 5 Centre for Integrated Research (CIR), Area of Diagnostic Imaging, Università “Campus Bio-Medico di Roma”, Rome, Italy; 6 Deptartment of Psychiatry and Mental Health, University of Cape Town, Cape Town, South Africa; Istituto Di Ricerche Farmacologiche Mario Negri, ITALY

## Abstract

Few studies have used diffusion tensor imaging (DTI) to investigate the micro-structural alterations of WM in patients with restrictive eating disorders (rED), and longitudinal data are lacking. Twelve patients with rED were scanned at diagnosis and after one year of family-based treatment, and compared to twenty-four healthy controls (HCs) through DTI analysis. A tract-based spatial statistics procedure was used to investigate diffusivity parameters: fractional anisotropy (FA) and mean, radial and axial diffusivities (MD, RD and AD, respectively). Reduced FA and increased RD were found in patients at baseline in the corpus callosum, corona radiata and posterior thalamic radiation compared with controls. However, no differences were found between follow-up patients and controls, suggesting a partial normalization of the diffusivity parameters. In patients, trends for a negative correlation were found between the baseline FA of the right anterior corona radiata and the Eating Disorder Examination Questionnaire total score, while a positive trend was found between the baseline FA in the splenium of corpus callosum and the weight loss occurred between maximal documented weight and time of admission. A positive trend for correlation was also found between baseline FA in the right anterior corona radiata and the decrease in the Obsessive-Compulsive Inventory Revised total score over time. Our results suggest that the integrity of the limbic–thalamo–cortical projections and the reward-related circuitry are important for cognitive control processes and reward responsiveness in regulating eating behavior.

## Introduction

Restrictive eating disorders (rED), such as anorexia nervosa (AN) restrictive type and restrictive OSFED (Other Specified Feeding or Eating Disorder; [1]) are associated with changes in grey matter (GM) and white matter (WM) volumes both globally and regionally [2, 3]. However, few studies have used diffusion tensor imaging (DTI) to investigate the micro-structural alterations of WM in rED patients [4]. DTI allows the study of WM diffusion parameters, namely fractional anisotropy (FA) and mean, radial and axial diffusivity (respectively MD, RD and AD), which are reflective of the micro-structural integrity for WM. FA reflects the WM fibers’ density, while the RD reflects the integrity of the myelin sheaths [5, 6] and axonal density [7], and the AD reflects axonal damage [5, 6]. Taken together, these measures provide unique information on the structural connectivity of the brain [8]. The few DTI studies available in patients with EDs have mostly focused on adult patients with anorexia nervosa (AN) [9–14]. These studies have reported altered diffusion parameters mostly in the fornix [9–12], in the fronto-occipital tracts [9, 10, 12] and in the cingulum [9, 12, 13].

DTI information regarding adolescent patients with rED are scarce, with three studies currently available [15–17], only one of which also provided exploratory longitudinal information on WM micro-structural changes over time [17]. These studied focused on patients with AN, and two out of three also included non-restrictive AN, admitting also patients diagnosed with a binge eating/purging subtype. This might partially explain the different patterns of WM involvement reported by these studies. A cross-sectional DTI study focusing on the restrictive subtype demonstrated lower FA in the fimbria-fornix, in the corpus callosum and in the right superior longitudinal fasciculus (SLF), and increased FA in the left SLF [15]. Another cross-sectional study reported reduced FA in the fornix and in posterior frontal and parietal areas, but increased FA in the anterior frontal, orbitofrontal, and temporal lobes [16]. A recent longitudinal study in AN adolescent patients investigated changes in DTI parameters occurring over time [17]. In this study, twenty-two patients were enrolled at baseline, nine of whom were re-evaluated after weight rehabilitation. Increased FA was found in twenty-two patients at admission in frontal, parietal and temporal areas [17]. The FA values appeared to normalize after weight recovery [17]. This study, however, also included patients with binge eating/purging subtype and some of them had psychiatric comorbidities at admission. In partial contrast, a recent longitudinal study in a larger sample of 22 patients reported the persistence of altered FA values in the fronto-accumbal circuitry after weight restoration in patients diagnosed with AN [18]. However, in this study both adolescent and adult patients were included, and the analysis was *a priori* restricted to the fronto-accumbal circuitry.

Our study is, to the best of our knowledge, the first study to explore the longitudinal changes in WM integrity in a sample of adolescents including only rED patients, and the first DTI study to investigate WM micro-structural alterations in rED other than AN, such as OSFED—formerly EDNOS (eating disorder not otherwise specified) from DSM-IV [19]). Indeed, our sample consisted mostly of OFSED, which are the most prevalent amongst adolescents [20]. We studied 12 newly-diagnosed patients and 24 healthy controls (HCs) matched for age and sex through a DTI analysis, providing novel information on WM alterations in rED adolescents. Patients were re-evaluated after one year of family-based treatment. The longitudinal design of our study allowed us to investigate whether an early treatment of rEDs could be effective in reversing WM diffusivity alterations.

## Methods

### Participants

All participants gave written consent to participate in the study, and the protocol was approved by the Regional Ethical Review Board in Uppsala. A written informed consent form was signed by both the patients and their parents. Twelve female patients diagnosed with a rED were enrolled by the Eating Disorder Unit of the Department of Child and Adolescent Psychiatry at Uppsala Hospital, Sweden. The patients were scanned upon diagnosis (mean age: 15 years, age range: 13–17 years) and after one year of treatment [21, 22]. The scans were acquired between May 2011 and April 2015. One patient fulfilled the diagnostic criteria for AN according to the DSM-IV, while eleven were diagnosed as EDNOS. All patients were also recoded according to the new DSM-5 diagnostic criteria; two patients then fulfilled the criteria for AN, while ten patients were diagnosed as OSFED. Demographic data, as well as diagnoses according to the DSM-IV and DSM-5, are reported in Table 1. All patients underwent an MRI scanning session at diagnosis and after one year of family-based treatment. Twenty-four female HCs (mean age: 14 years) were also enrolled, and were scanned once at baseline.

**Table 1:** Clinical and demographic data of the patients.

Baseline							Follow-up				
Pt	Age	BMI (Kg/m^2^)	Weight loss (Kg)	BMI SDS	Menstruations	DSM-IV/ DSM-5 diagnosis	Diagnosis during treatment	Weight increase (Kg)	BMI (Kg/m^2^)	BMI SDS	DSM-IV/DSM-5 diagnosis
1	15	20.0	4.0	0.1	secondary amenorrhea	EDNOS/OSFED	*Depression*	4.0	21.3	0.4	no ED/no ED
2	15	15.7	5.2	-2.4	menstruations	EDNOS/AN	*None*	7.2	18.2	1.2	EDNOS/OSFED
3	17	17.8	10.5	-1.7	secondary amenorrhea	EDNOS/OSFED	*Depression*	6.1	19.8	-0.7	no ED/no ED
4	17	21.3	15.8	0.2	menstruations	EDNOS/OSFED	*Depression*, *suspect of ADHD*	1.4	21.4	0.0	EDNOS/OSFED
5	13	18.4	1.4	-0.2	menstruations	EDNOS/OSFED	*Depression*, *ADHD*, *PTSD*	-1.5	17.6	-1.0	EDNOS/OSFED
6	16	21.2	5.1	0.3	hormonal contraceptives	EDNOS/OSFED	*Depression*, *PTSD*, *OCD*	8.1	23.3	0.9	no ED/no ED
7	13	13.5	9.0	-0.4	premenarche	EDNOS/OSFED	*Depression*, *anxiety disorder*	13.3	18.0	-0.5	no ED/no ED
8	16	16.6	21.8	-2.0	secondary amenorrhea	AN/AN	*None*	19.2	23.9	1.1	no ED/no ED
9	13	17.9	missing	-0.9	menstruations	EDNOS/OSFED	*None*	6.4	20.2	0.2	no ED/no ED
10	14	24.9	2.4	1.7	menstruations	EDNOS/OSFED	*None*	5.2	26.8	2.1	no ED/no ED
11	17	18.5	7.7	-1.2	menstruations	EDNOS/OSFED	*None*	5.3	20.3	-0.5	no ED/no ED
12	14	18.1	12.6	-0.7	secondary amenorrhea	EDNOS/OSFED	*None*	12.0	22.2	0.8	EDNOS/OSFED

BMI: Body Mass Index; SDS: Standard Deviation Score; DSM: Diagnostic and Statistical Manual of Mental Disorders

BMI was calculated, as well as self-reported measures of ED-related and obsessive-compulsive symptoms, through the administration of the Eating Disorder Examination Questionnaire (EDE-Q) and the Obsessive-Compulsive Inventory Revised (OCI-R), respectively. The EDE-Q consists of four subscales: restraint, weight concern, eating concern, and shape concern [23]. For each item the score range from 0, corresponding to "no days", to 6, corresponding to "every day". The OCI-R is a shorter version of the OCI and includes six subscales: washing, checking, ordering, obsessing, hoarding and neutralizing [24]. For each item, the scores range from 0 ("not at all") to 4 ("extremely"). Groups mean BMI, and EDE-Q and OCI-R total scores are reported in Table 2.

**Table 2:** Groups characteristics.

	Patients at baseline (mean)	Controls at baseline (mean)	Patients at follow-up (mean)
*Age (years)*	15.3	14.1	16.4
*BMI (Kg/m*^*2*^*)*	18.7	20.6	21.1
*EDE-Q total score*	3.7	0.8	2.1
*OCI-R total score*	35.6	9.4	25.9

BMI: body mass index; EDE-Q: Eating Disorder Examination Questionnaire; OCI-R: Obsessive-compulsive Inventory Revised

Exclusion criteria were male sex, comorbidity with neurological diseases, lefthandedness, metallic implants, claustrophobia and use of psychotropic medication. HC had no history of psychiatric conditions, and none of the patients had psychiatric comorbidities when recruited.

### Treatment

All patients underwent one year of family-based out-patient treatment [22]. In this approach, the role of the parents in the care of their adolescent is emphasized. The first phase aims to reestablish a normal meal routine and stop weight loss, and until these goals are reached, school attendance is not allowed. Parents are suggested to serve three main meals and two snacks a day and should eat all meals together with the adolescent. They should also prevent the adolescent from using compensatory behaviors such as vomiting or exercising. The second phase aims to restore weight gain, with a rate of 0.5–1 Kg per week. Frequent visits to the hospital, as well as at-home visits from health care professionals, are planned. The third phase consists of a gradual reintroduction to school. In the fourth phase, patients with ongoing difficulties with social relations and self-esteem are offered further treatment [21]. Treatment is goal oriented and not set to a fixed number of sessions. Details of the treatment have been published elsewhere [22].

### MRI acquisition

A Philips 3-Tesla scanner (Achieva, Philips Healthcare, Best Netherlands) using a standard 32-channel head coil was used to acquire the MRI sequences. Diffusion tensor imaging data were acquired using an echoplanar imaging sequence (EPI) (TR: 6700 ms, TE: 77 ms, voxel size: 1.75x1.75x1.75 mm^3^, 48 directions, 60 axial slices covering the whole brain).

### Pre-processing

All pre-processing steps were carried out in FMRIB Software Library (FSL, provided in the public domain by the Oxford Centre for Functional Magnetic Resonance Imaging of the Brain) [25]. DTI images were first corrected for eddy currents and head motion using the FMRIB’s Diffusion Toolbox (FDT) implemented in FSL, then brain images were extracted using the brain extraction tool (BET) [26]. The diffusion tensor model was then fitted at each voxel, obtaining FA, MD, and AD maps. The second and third eigenvalues were then averaged to obtain the RD maps.

### Tract-based spatial statistics

A whole-brain, voxel-wise Tract-based Spatial Statistics (TBSS) [27] analysis was performed using the FSL. TBSS procedure allows to overcome some of the limitations related to the registration algorithms and the need for spatial smoothing required by the voxel-based morphometry techniques [8]. One outlier was detected (one patients at follow-up) and excluded from further analysis.

For each analysis, the FA images were aligned to a common target (FMRIB58_FA; http://fsl.fmrib.ox.ac.uk/fsl/fsl4.0/tbss/FMRIB58_FA.html) in the MNI 152 standard space using nonlinear registration, and resampled to a 1x1x1 mm^3^ voxel size. The normalization parameters were then applied to the other diffusivity parameters maps (AD, RD, MD). FA images were averaged to create a mean FA image, then a skeleton was created, representing the center of all fibers bundle, using a threshold of FA > 0.2 to exclude voxels not belonging to WM. FA maps of each participant were then projected onto the skeleton. The same procedure was applied to the MD, RD and AD maps.

### Statistical analysis of DTI data

Statistical analyses were carried out using a non parametric permutation-based statistics [27] in FSL. Differences in FA were first tested between baseline patients and controls, entering age as a covariate of no interest in the analysis. The number of permutations was set at 10000, and the threshold for significance was set at p < 0.05, corrected for multiple comparisons at cluster level with a threshold-free cluster enhancement (TFCE) approach [28]. When an altered FA value was detected, RD, MD and AD were also tested. For each of the 48 structures reported in the ICBM-DTI-81 white-matter labels atlas provided by John Hopkins University and implemented in FSL, the extent of involvement was calculated and is reported in the results Tables 3 and 4.

**Table 3:** Fractional anisotropy baseline assessment.

Extent (vox)	Side	Structure
811		Genu of corpus callosum
584		Body of corpus callosum
261		Splenium of corpus callosum
609	R	Anterior corona radiata
537	L	Anterior corona radiata
598	R	Superior corona radiata
270	L	Superior corona radiata
315	R	Posterior corona radiata
121	R	Posterior thalamic radiation
27	R	Tapetum

**Table 4:** Radial diffusivity baseline assessment.

Extent (vox)	Side	Structure
772		Genu of corpus callosum
1049		Body of corpus callosum
286		Splenium of corpus callosum
426	R	Anterior corona radiata
310	L	Anterior corona radiata
566	R	Superior corona radiata
272	L	Superior corona radiata
273	R	Posterior corona radiata
24	L	Posterior corona radiata
58	R	Posterior thalamic radiation
20	R	Tapetum

Longitudinal analyses between patients at baseline and patients at follow-up, and between patients at follow-up and controls at baseline, were masked for the supra-threshold clusters from the baseline analysis (baseline patients vs controls), to investigate the progress of the detected diffusivity alterations over time. The number of permutations was again set at 10000, and the threshold for significance was set at p < 0.05, corrected for multiple comparisons at cluster level with a TFCE approach [28]. When an altered FA value was detected, RD, MD and AD were tested. Age was entered as a covariate in the analysis. However, given the lack of longitudinal controls, in order to further explore whether the age difference between baseline and follow-up could be influencing the results of the analysis, Spearman’s coefficient was calculated to test for correlations between FA values in the involved structures and age. This correlation analysis was performed using Statistical Package for Social Science (SPSS; http://www-01.ibm.com/software/it/analytics/spss/), after extracting the FA values from the selected structures. The significance threshold was set at p < 0.05, corrected for multiple comparisons with a False Discovery Rate approach [29].

### Clinical-imaging correlations

Clinical-imaging correlation analyses were performed with SPSS. The FA values of the structures where diffusivity was found to be altered between patients and controls were extracted. At baseline, Spearman’s correlation coefficient was calculated to test for correlations between BMI, EDE-Q and OCI-R total scores and FA values, separately in patients and controls. Within the baseline patients, the FA values were also tested for correlations with the weight loss between the maximum documented weight and the time of admission. Moreover, baseline FA in patients was tested for correlations with the percentage increase in BMI and the percentage decrease of the total scores on the EDE-Q and OCI-R questionnaires after treatment. The threshold for significance was set at p<0.05, corrected for multiple comparisons with a FDR approach.

## Results

### Reduced fractional anisotropy and increased radial diffusivity in patients at baseline

**Fig 1:**
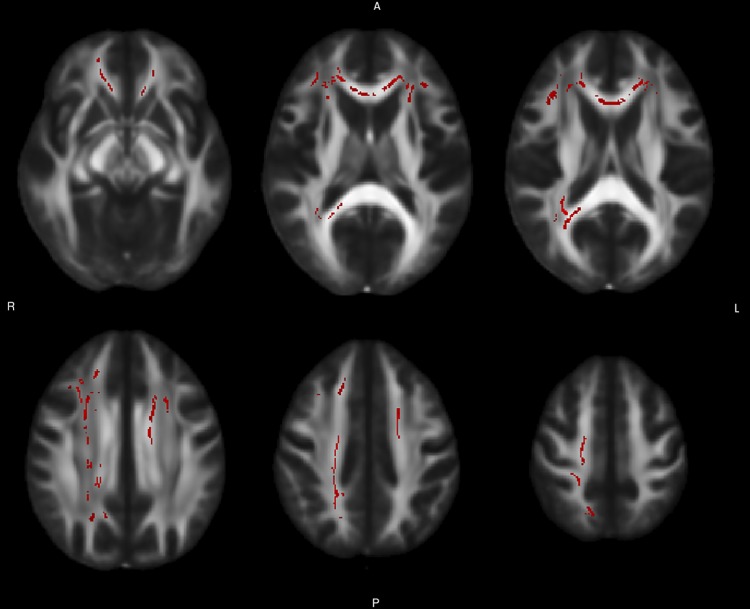
Baseline differences in fractional anisotropy between patients and controls. The figure reports axial slices showing the tracts of WM where a significant difference in fractional anisotropy was found between baseline patients and controls at the TBSS analysis. A permutation-based test was used, with the number of permutation set at 10000. The threshold for significance was set at p < 0.05, corrected for multiple comparison with a threshold-free cluster enhancement approach. The WM tracts are superimposed to the FMRIB58_FA standard provided with FSL.

**Fig 2:**
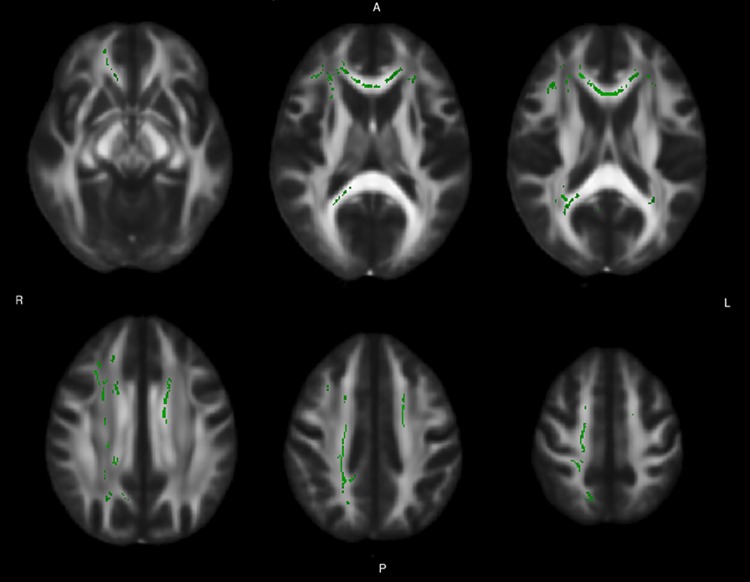
Baseline differences in radial diffusivity between patients and controls. The figure reports axial slices showing the tracts of WM where a significant difference in radial diffusivity, calculated as the mean of the second and third eigenvalues, was found between baseline patients and controls at the TBSS analysis. A permutation-based test was used, with the number of permutation set at 10000. The threshold for significance was set at p < 0.05, corrected for multiple comparison with a threshold-free cluster enhancement approach. The WM tracts are superimposed to the FMRIB58_FA standard provided with FSL.

Reduced FA was found in patients at baseline, compared with controls, in the genu, body and splenium of corpus callosum, in the anterior and superior corona radiata bilaterally, in the right posterior corona radiata and posterior thalamic radiation, and in the right tapetum (Fig 1, Table 3). Radial diffusivity was increased in the same structures, with the additional involvement of a small cluster in the left posterior corona radiate (Fig 2, Table 4). No alterations were found in the AD or MD values.

### Within-patients longitudinal analysis

**Fig 3:**
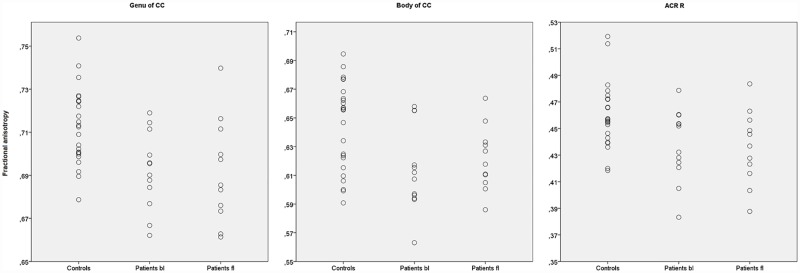
Between-groups differences in FA values. The scatterplots report the FA values of the structures were differences were found between the patients at baseline and controls. The three most extensively involved structure are reported. CC = Corpus Callosum, ACR = Anterior Corona Radiata, bl = baseline, fl = follow-up.

No differences were found between the baseline and follow-up patients, nor between follow-up patients and baseline controls, in FA values. Scatterplots of FA values in patients at baseline, patients at follow-up and controls are reported for structures were reduced FA was found at baseline (Fig 3). No correlations emerged between age and FA values in the structures selected for the analyses.

### Fractional anisotropy correlations with clinical scores at baseline

Within baseline patients, a negative correlation was found between the EDE-Q total score and the FA in the right anterior corona radiata (p < 0.02). This correlation was not present in the control group. Moreover, a negative correlation was found between the FA in the splenium of corpus callosum and the weight loss (p < 0.02) between maximal documented weight and admission. In HCs, a negative correlation was found between the BMI and the FA in the genu (p < 0.03) and body (p < 0.02) of corpus callosum, and in the left anterior corona radiata (p < 0.03).

None of these correlations, however, survived the false discovery rate (FDR) correction for multiple comparisons. A summary of the correlations is reported in Table 5.

**Table 5:** Clinical-imaging correlations.

***Patients at baseline***			
**Clinical variables**	**WM structure**	**Spearman's rho**	**p**
EDE-Q	Right anterior corona radiata	-0.673	0.02
OCI-R % decrease	Right anterior corona radiata	-0.667	0.05
Weight loss	Splenium of corpus callosum	-0.700	0.02
***Controls***			
**Clinical variables**	**WM structure**	**Spearman's rho**	
BMI	Genu of corpus callosum	-0.455	0.03
BMI	Body of corpus callosum	-0.458	0.02
BMI	Left anterior corona radiata	-0.443	0.03

### Fractional anisotropy correlations with clinical scores improvement

In patients, the FA in the right anterior corona radiata at baseline showed a positive correlation with the decrease in the OCI-R total score over time (p < 0.05), not surviving the correction for FDR (Table 5).

## Discussion

This is, to the best of our knowledge, the first study to investigate WM integrity through DTI analysis in a sample of adolescent patients with purely restrictive EDs, and the first one to provide information on longitudinal changes occurring in WM diffusivity parameters over time. We studied 12 patients and 24 healthy controls, and found that, compared with controls, patients had reduced FA in corpus callosum, corona radiata, and right posterior thalamic radiation and tapetum. The FA reduction was mostly due to an increased RD, mainly reflective of alterations in the mylien sheath of the WM fibers [5, 6], though also sensitive to changes in axonal diameters or density [7]. Such alterations were undetectable when comparing the patients at follow-up with controls, suggesting a partial normalization of the diffusivity parameters. Furthermore, in baseline patients, the FA in the right anterior corona radiata showed a trend for a negative correlation with the EDE-Q global score, and the baseline FA in the splenium of corpus callosum showed a negative trend with the weight loss between maximal documented weight and time of admission. The improvement on clinical measures during treatment also showed a trend for correlation with the FA at baseline. In particular, a positive trend was found between the FA in the right anterior corona radiata at baseline and the decrease in the OCI-R total score over time.

The corpus callosum is a fundamental structure for inter-hemispheric connections and motor functions, and is suggested to be also involved in taste processing [30] through the connection between the primary gustatory cortices, tactile and somatosensory cortices [30]. The alterations in corpus callosum WM micro-structure, as reflected by the decreased FA and increased RD, might therefore impair the somatosensory integration regarding food. Moreover, the FA values in the corpus callosum have been found to positively correlate with reward-related activity in the striatum [31]. In particular, it has been hypothesized that the corpus callosum might influence the intensity of reward responsiveness of the ventral striatum, by regulating the efficiency in information transfer within reward-related circuitries [31]. We found a negative relationship between the corpus callosum FA and BMI in controls, a finding which has been previously reported in literature [32]. Interestingly, in patients the FA of the corpus callosum did not correlate with BMI nor with maximal document weight, but with the weight loss between the maximal documented weight and the time of clinical evaluation. Indeed, a peculiar feature of the OSFED patients is the importance of their weight loss, although the BMI at diagnosis is often in normal range.

The corona radiata conveys fibers from multiple cortical regions to basal ganglia and brain-stem structures, and is part of the limbic–thalamo–cortical circuitry [33–35]. In our sample, we found a reduced FA and increased RD in the superior, posterior and anterior part of the corona radiata. The corona radiata has been involved in central taste disorders [36], as projections from the somatosensory nerves from the lips and tongue run through the posterior part of corona radiata [36]. The anterior corona radiata conveys thalamic projections from the internal capsule to the prefrontal cortex areas, which are involved in reward seeking [37] and cognitive control over eating [38] and are central in the pathogenesis of rED [39–42]. In our sample, the FA in the right anterior corona radiata showed a trend toward a negative correlation with the ED-related symptoms in patients. This suggests that the integrity of the limbic–thalamo–cortical circuitry projections is critical for the adequate functioning of the prefrontal cortex, and its alteration might lead to a disruption of cognitive control processes. Alterations in the anterior corona radiata WM has also been reported in obsessive-compulsive disorder (OCD) [43]. Accordingly, we found the FA of the anterior corona radiata to be related with the improvement in the obsessive-compulsive symptoms over time.

The posterior thalamic radiation contains bidirectional thalamo-cortical projections connecting the thalamic nuclei with basal ganglia and visual, somatosensory, auditory and gustatory cortices, as well as pre-frontal areas. This structure is involved in cognitive control and attentional processes [44], and has been found to be altered in AN [14]. The reduction in FA values we found in the posterior thalamic radiation further corroborates the hypothesis that the gustatory pathway and the projections to prefrontal and reward-related regions in determining an alteration in food-related cognitive processing in adolescents, leading to a restrictive eating behavior.

The alterations in FA values appeared to revert after one year of follow-up. This suggests that a prompt intervention might be effective in arresting the disruption of WM micro-structural integrity that occurs even the first phases of the ED. An early treatment is of particular importance in adolescents, as during adolescence executive function structuring and neuro-plasticity processes are at their maximum [45, 46].

We did not find increased FA values in any WM tracts, in contrast to previous studies. However, the DTI studies available in adolescents focused on a sample consisting only of AN patients [15–17], thus not including other rED such as EDNOS/OSFED. Moreover, two of these studies also included non-restrictive subtypes of AN (i.e. binge eating/purging subtype) [16, 17]. Travis K.E. et al. [15], in particular, proposed that the pattern of increased RD coupled to decreased/increased FA they reported could be related to a reduction in the number of crossing fibers, and to an alteration in WM micro-structure due to a reduction in the myelin content caused by weight loss and starvation [15]. As AN patients typically have a lower body weight compared to OSFED patients, this may explain the lack of FA increase in our sample.

## Limitations

Some limitations to our study must be noticed. Ours is the largest adolescent cohort enrolled in a DTI longitudinal study in the field of rED so far, however our sample is still small. The lack of longitudinal controls, given the dynamic development of the brain during adolescent, calls for future studies to further confirm our findings. A longitudinal control group would also allow to rule out the presence of potential artifacts due to systematic signal drifts, and to detect minor effects that might be masked by inter-subject variance in the current analysis.

## Conclusions

This is the first study to explore the longitudinal changes in WM integrity in a sample of adolescents with rED, and the first DTI study to investigate WM micro-structural alterations in rED other than AN. Twelve patients and twenty-four controls were enrolled. Patients were scanned at diagnosis and after 1 year of family-based treatment. A TBSS procedure was performed on DTI data, and diffusivity parameters were calculated in a whole-brain, voxel-wise analysis. Reduced FA was detected at baseline in corpus callosum, corona radiata, and right posterior thalamic radiation and tapetum. The same structures showed an increased RD, indicative of alterations in WM microstructure. Such alterations were undetectable when comparing the patients at follow-up with controls, suggesting a normalization of the diffusivity parameters. Baseline FA also showed a trend for correlations with baseline clinical scores and with their changes after treatment. These structures are part of a limbic-thalamo-cortical circuitry comprising the gustatory pathway and the projections to prefrontal areas and reward-related regions. The reduced integrity of these pathways may contribute to the alteration in food-related cognitive processing present in adolescents with rED. Future studies in larger samples may help further elucidate the role WM integrity in the pathogenesis of rED.
