# Correction: Limbic-thalamo-cortical projections and reward-related circuitry integrity affects eating behavior: A longitudinal DTI study in adolescents with restrictive eating disorders

**DOI:** 10.1371/journal.pone.0176646

**Published:** 2017-04-20

**Authors:** Gaia Olivo, Lyle Wiemerslage, Ingemar Swenne, Christina Zhukovsky, Helena Salonen-Ros, Elna-Marie Larsson, Santino Gaudio, Samantha J. Brooks, Helgi B. Schiöth

The fourth author’s name is spelled incorrectly. The correct spelling is Christina Zhukovsky. The correct citation is:

Olivo G, Wiemerslage L, Swenne I, Zhukovsky C, Salonen-Ros H, Larsson E-M, et al. (2017) Limbic-thalamo-cortical projections and reward-related circuitry integrity affects eating behavior: A longitudinal DTI study in adolescents with restrictive eating disorders. PLoS ONE 12(3): e0172129. doi:10.1371/journal.pone.0172129
